# Supplementary material for: Correlation between topological band character and chemical bonding in a Bi14Rh3I9-based family of insulators
Source: Sci Rep. 2016 Feb 15;6:20645. doi: 10.1038/srep20645 (PMC4753431; doi:10.1038/srep20645)
Supplement: Supplementary Information [file srep20645-s1.pdf]

Supplementary Information

Correlation between topological band character and chemical bonding in a $Bi_{14}Rh_3I_9$-based family of insulators

Bertold Rasche[1,*], Anna Isaeva[1], Michael Ruck[1,2], Klaus Koepernik[3], Manuel Richter[3], and Jeroen van den Brink[3,+]

[1]Department of Chemistry and Food Chemistry, TU Dresden, D-01062 Dresden, Germany
[2]Max Planck Institute for Chemical Physics of Solids, D-01187 Dresden, Germany
[3]Leibniz Institute for Solid State and Materials Research, IFW Dresden, D-01069 Dresden, Germany

Structural Modelling

The overall structural complexity of $Bi_{14}Rh_3I_9$ can be reduced by recovering the symmetry of the QSH layer $[(Bi_4Rh)_3I]^{2+}$. In the resulting model, a single QSH layer is isolated and sandwiched between iodide ions that substitute for the $[Bi_2I_8]^{2-}$ spacers. This isolated-layer structure (ILS) has the hexagonal layer symmetry $p6/mmm$ (Figure 1). We compensated the changes in the total charge of the QSH layer that are induced by the modification of the spacer layer by reducing the charges of the spacer iodide ions down to -0.25 via VCA: $[({}_{53.75}I)_4]^-[(Bi_4Rh)_3I]^{2+}[({}_{53.75}I)_4]^-$. Here, the notation ${}_ZI$ means an iodine-like pseudo-atom with the nuclear charge Z. The resulting Rh-ILS was relaxed using the FPLO program package under the restricted $p6/mmm$ layer symmetry and by varying the cell axes with a step width of 0.1Å. All atomic positions were optimised on each step with a convergence criterion of 10^{-3}eV/Å. The same procedure was used for all other model structures discussed in this paper.

Already the fact that the density-of-states (DOS) in the original compound $Bi_{14}Rh_3I_9$ close to the Fermi-level is dominated by the DOS of the QSH-layer is a strong support for the creation of an ILS. Furthermore, comparing the features of the original DOS and the ILS-DOS shows very good agreement (Figure S1). Direct comparison of the electronic band-structure of the ILS with the one of the parent compound $Bi_{14}Rh_3I_9$ is achieved by unfolding onto a hexagonal BZ (Figure S2). The unfolded bands close to the Fermi-level can easily be identified in the band-structure of the hexagonal Rh-ILS (Figure S2). Also the topological character of the various band gaps carries over directly to the ILS, in spite of a slight relative shift of the bands resulting in a small increase of the lower topological gap at the expense of the upper one. This confirms that the influence of the spacer as well as of the stacking of the QSH layers on the electronic structure is small. Having established a reliable method to model the essentials of the $Bi_{14}Rh_3I_9$ electronic structure, we now replace Rh by the other platinum group elements (Ru, Os, Ir, Pd, Pt) in the QSH layer, keeping the number of valence electrons the same via the VCA and calculate the electronic structure and the topological invariants for the corresponding M-ILSs. The atomic distances are almost the same for all relaxed M-ILSs (Figure S3).

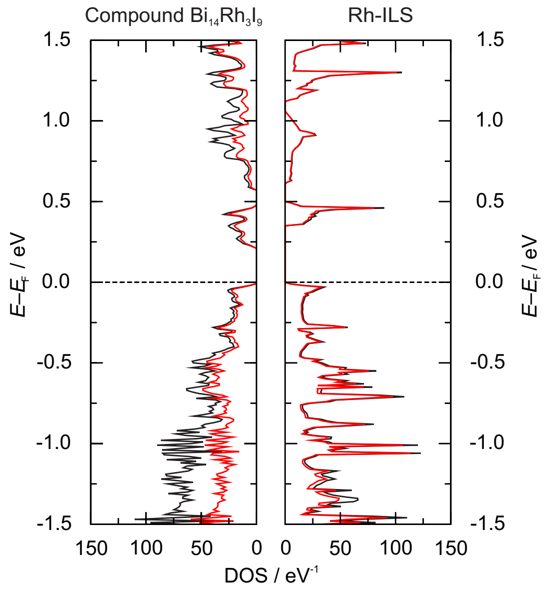

Figure S1. Total density of states (DOS, black) for the original compound $Bi_{14}Rh_3I_9$ (left) and the isolated layer model (right) with the partial DOS for the intermetallic layer (red).

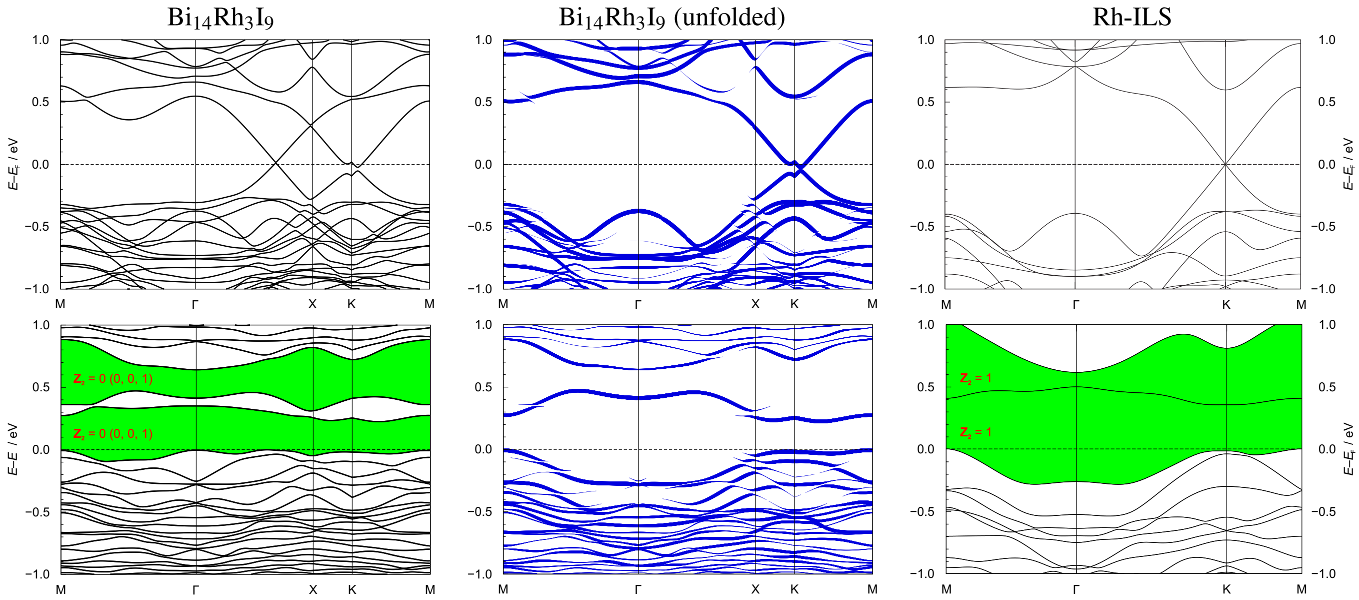

Figure S2. Electronic band-structure and unfolded electronic band-structure for $Bi_{14}Rh_3I_9$ (left) compared to the electronic band-structure of the model (right), in a scalar-relativistic (upper line) and a full-relativistic approach (lower line), respectively. For the gap at and above the Fermi-energy the topological invariants are given.

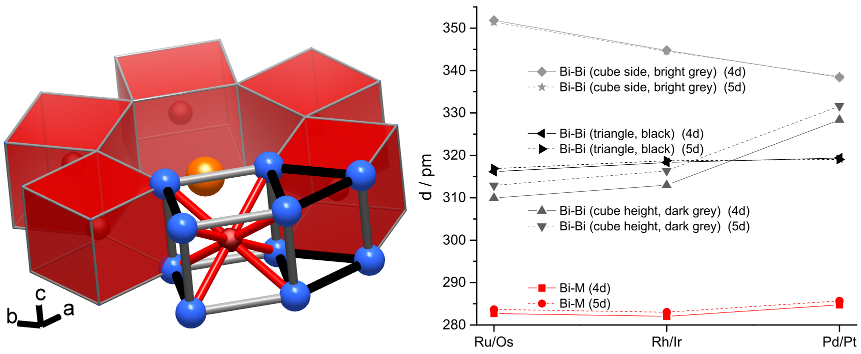

Figure S3. Bond lengths for the Bi-M bonds and all Bi-Bi bonds in the ILSs.

Electronic Structure

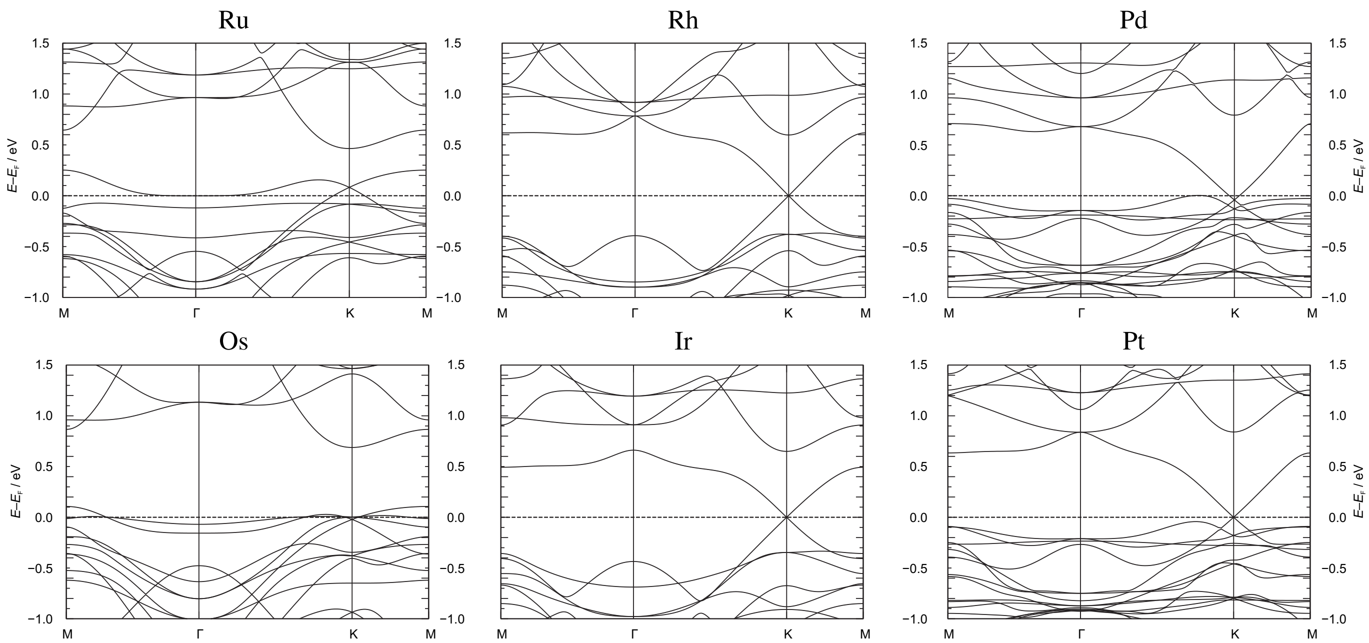

Figure S4. Scalar-relativistic band-structures for the ILS-series, all with a valence-electron count equal to the one in the original QSH-layer $[(Bi_4Rh)_3I]^{2+}$, arranged as in the periodic table of elements.

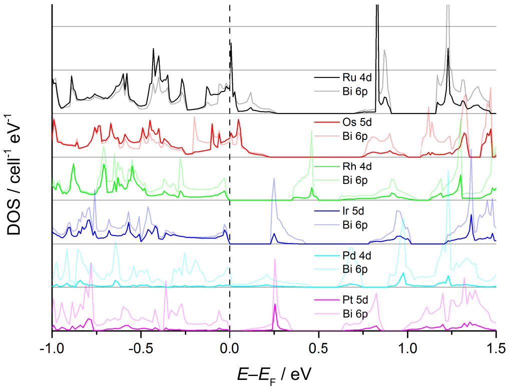

Figure S5. Partial DOS of the transition element d-states and the bismuth 6p-states for the six models with a valence-electron count equal to the one in the original QSH-layer $[(Bi_4Rh)_3I]^{2+}$.

Ru

Band #	Γ	M	M	M	Z_2
539	-1	-1	-1	-1	0
541	1	1	1	1	0
543	-1	1	1	1	1
545	-1	1	1	1	1
547	-1	1	1	1	1
549	-1	-1	-1	-1	0
551	-1	1	1	1	1
553	-1	-1	-1	-1	0
555	-1	-1	-1	-1	0
557	-1	1	1	1	1
559	1	1	1	1	0
561	1	1	1	1	0
563	1	-1	-1	-1	1
565	1	-1	-1	-1	1

Rh

Band #	Γ	M	M	M	Z_2
539	-1	-1	-1	-1	0
541	1	-1	-1	-1	1
543	-1	1	1	1	1
545	1	1	1	1	0
547	1	-1	-1	-1	1
549	-1	-1	-1	-1	0
551	1	1	1	1	0
553	1	-1	-1	-1	1
555	-1	-1	-1	-1	0
557	-1	1	1	1	1
559	-1	1	1	1	1
561	1	1	1	1	0
563	1	-1	-1	-1	1
565	1	1	1	1	0

Pd

Band #	Γ	M	M	M	Z_2
539	-1	-1	-1	-1	0
541	1	-1	-1	-1	1
543	1	1	1	1	0
545	1	1	1	1	0
547	-1	-1	-1	-1	0
549	1	-1	-1	-1	1
551	1	1	1	1	0
553	-1	-1	-1	-1	0
555	1	-1	-1	-1	1
557	-1	1	1	1	1
559	-1	1	1	1	1
561	1	-1	-1	-1	1
563	1	-1	-1	-1	1
565	1	1	1	1	0

Os

Band #	Γ	M	M	M	Z_2
581	-1	1	1	1	1
583	-1	1	1	1	1
585	1	-1	-1	-1	1
587	1	-1	-1	-1	1
589	-1	1	1	1	1
591	1	-1	-1	-1	1
593	-1	-1	-1	-1	0
595	1	-1	-1	-1	1
597	1	1	1	1	0
599	1	-1	-1	-1	1
601	-1	-1	-1	-1	0
603	-1	-1	-1	-1	0
605	-1	1	1	1	1
607	-1	1	1	1	1

Ir

Band #	Γ	M	M	M	Z_2
581	1	1	1	1	0
583	1	-1	-1	-1	1
585	-1	-1	-1	-1	0
587	1	-1	-1	-1	1
589	1	1	1	1	0
591	1	1	1	1	0
593	1	-1	-1	-1	1
595	1	1	1	1	0
597	1	1	1	1	0
599	-1	1	1	1	1
601	-1	-1	-1	-1	0
603	-1	-1	-1	-1	0
605	-1	-1	-1	-1	0
607	-1	1	1	1	1

Pt

Band #	Γ	M	M	M	Z_2
539	-1	-1	-1	-1	0
541	1	-1	-1	-1	1
543	1	1	1	1	0
545	-1	1	1	1	1
547	1	-1	-1	-1	1
549	1	-1	-1	-1	1
551	1	1	1	1	0
553	-1	-1	-1	-1	0
555	1	-1	-1	-1	1
557	-1	1	1	1	1
559	-1	1	1	1	1
561	1	1	1	1	0
563	1	-1	-1	-1	1
565	1	1	1	1	0

Table S1. Calculated Z_2 invariants at the four time-reversal-inversion-invariant k-points (in two dimensions) for all ILSs with a valence-electron count equal to the one in the original QSH-layer $[(Bi_4Rh)_3I]^{2+}$ (blue: filled band; light blue: partially filled band; orange: empty band). Energy gaps are marked with a black line.

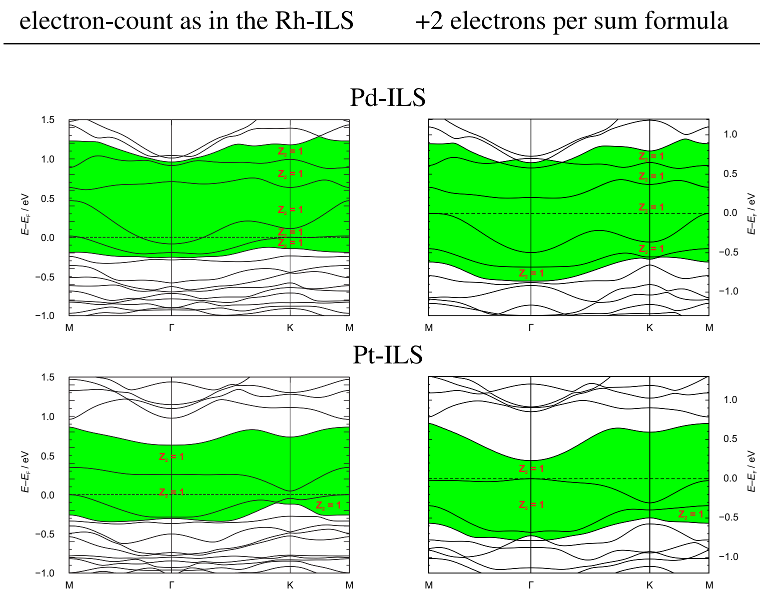

Figure S6. Full-relativistic band-structures for the Pd- and Pt-ILS for different valence electron counts in the intermetallic layer with the calculated 2D-Z_2 invariants for gaps close to the Fermi-energy.

Origin of the topological nature

The tight-binding description

The essential parts of the Bi-M-I system can be understood by turning on interactions step by step.

For this purpose a minimal tight-binding (TB) model was constructed, which contains the Bi-couplings between Bi atoms in the triangle and between the triangles (the in-cube hopping), the Bi bilayer hopping in vertical direction, the Rh-Rh direct interaction and the NN Bi-Rh hopping. With this minimal model, which already contains a sizeable number of parameters the scalar-relativistic band-structure of all 6 systems can be reproduced such that all important features around the Fermi level are reproduced, most important of which are the energy position of the Dirac-cone at the K-point and the formation of band crossings (second Dirac-cone) at the bottom of the conduction bands at the Γ-point. The TB-parameters of the model are derived from maximally projected Wannier functions obtained from scalar-relativistic FPLO calculations and are set-up with scaling factors to smoothly switch on various interactions.

The basic ingredient of the system are the planar networks of triangles and cubes formed by the Bi atoms. We first focus on the p_z orbitals, since those form the Dirac-cone at the K-point in graphene. It turns out that the in-triangle interaction (-0.5eV) is larger than the inter-triangle (in-cube) interaction (-0.3eV), which corresponds to the slightly larger Bi-Bi distance between triangles as compared to within the triangles. With only the Bi p_z interaction non-zero and without Bi-Bi bi-layer (BL) interaction the system has effectively 6 bands. These form two Dirac-cone like crossings at the K-point and two doubly degenerate band crossings at the Γ-point. If one thinks of the orbitals in each triangle as a single object one obtains a graphene lattice (of Bi-triangles). If all Bi p_z orbitals form a maximally symmetric linear combination (equal phase) the two resulting triangular molecular orbitals (MO) behave as the carbon p_z orbital would do. At the K-point a twofold degeneracy must occur, which leads to the scalar-relativistic Dirac point. To corroborate this picture we plot the band-structure resulting from the interaction set-up discussed above coloured by band weights of the triangular molecular orbital in Figure S7.

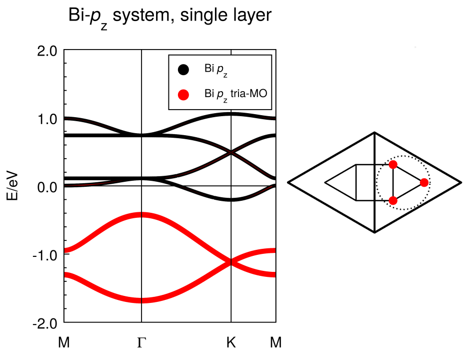

Figure S7. The Bi-p_z single layer system with band character of the triangular molecular orbital in red (light gray) and general p_z character in black. Right panel: a sketch of the triangular molecular orbital containing 3 p_z-orbitals of equal phase.

As can be seen the 6 bands split into two band complexes of 2 and 4 bands respectively of which the lower 2-band manifold has a dispersion very similar to the dispersion of the $2p_z$-bands in graphene, only that the MO of the three Bi p_z orbitals at the triangle corners replaces the carbon $2p_z$.

Now, we switch on the Bi p_z-Bi p_z bilayer coupling, which is of the order 1.9eV and hence much larger than the couplings, which form the single layer band-structure. We therefore essentially expect a doubling of the single-layer band-structure with a large gap. In our minimal model the inclusion of only vertical bi-layer coupling leads to an exact doubling without any further hybridisations. We again use the projection onto single-atom like molecular orbitals. This time however the MO consist of 6 atoms belonging to one of the two triangular Bi-prisms. Within the prism we can chose the upper three atoms to be in phase or in anti-phase to the lower three atoms, which basically corresponds to a bi-layer bonding (B)/anti-bonding (AB) molecular orbital (Figure S8).

What matters here is that later we will see that the bi-layer anti-bonding MO ends up at the Fermi energy and forms a Dirac-cone. This makes the connection to graphene more striking since the effective BL-AB MO has an all over p_z-like nature. (Although this is probably not necessary for the symmetry argument which leads to the cone at the K-point given that we have two such cones: B and AB.)

We extracted the TB parameters independently for all 6 systems. However, it turns out that they have rather similar sets of parameters. The differences, which turn out to be most significant are the onsite energies of the Bi p_z and $p_{x,y}$ orbitals and of the transition metal (M) d-orbitals. For the M onsite terms and for the $p_{x,y}$ orbitals the various onsite energies fall into a certain range with the energy order for the M d-orbitals being $d_{x^2-y^2} < d_{z^2} < d_{xy} < d_{yz,xz}$ (see Table S2). From the difference

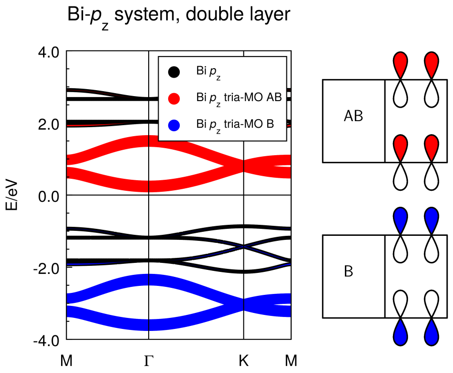

Figure S8. The Bi-p_z double layer system with bi-layer-bonding and anti-bonding triangular molecular orbital weights. The sketch shows a side view of the unit cell with a BL anti-bonding and bonding triangular MO.

between the p_z and d onsite energies one can conclude the order of compounds in terms of varying p_z-d separation: Os, Ru, Ir, Rh, Pt, Pd. The actual band-structures and the topological properties also follow this ordering scheme. (The order of Pd and Pt is not very conclusive.) For instance the energy position of the scalar-relativistic Dirac-cone at the K-point is determined by this sorting order of the compounds.

	$p_z - p_z$ BL	M d-onsite	Bi p_z-onsite	Bi p_{xy}-onsite
Ru	1.84	$-0.4\ldots-1.0$	-0.4	$-0.18\ldots0.0$
Rh	1.92	$-1.3\ldots-1.8$	-0.07	$-0.13\ldots0.07$
Pd	1.79	$-2.5\ldots-2.8$	0.07	$-0.18\ldots0.23$
Os	1.82	$-0.18\ldots-0.97$	-0.41	$-0.17\ldots0.06$
Ir	1.88	$-1.15\ldots1.82$	-0.18	$-0.15-0.06$
Pt	1.76	$-2.3\ldots-2.8$	0.0	$-0.18\ldots0.18$

Table S2. Most distinguishing terms in the TB-models.

Because of the similarity of the parameter sets it is possible to mimic the actually calculated models of the whole series of compounds using only the Rh-model simply by shifting the onsite energies of the d-orbitals relative to the Bi orbitals. There are small differences between the native model and the modified Rh-model but the features are certainly similar in both cases. After all we consider a minimal model only, which shows small deviations from the real band-structure calculations. This approach allows us to follow the development of the band-structure throughout the whole series by using just one model but with varying M d-orbital onsite energies. Note, that in the following we do not fix the Fermi level to account for the proper band filling. We only focus on the development of the band-structure with respect to varying parameters.

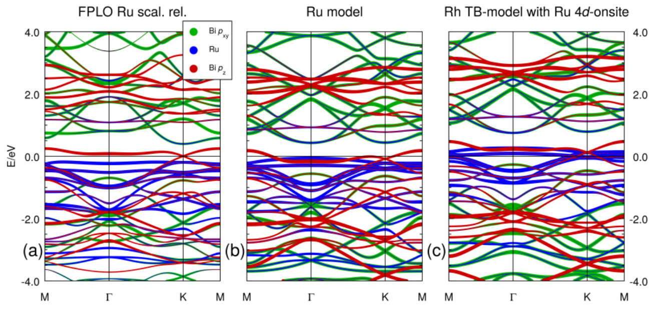

Figure S9. Comparison of (a) the actual scalar-relativistic FPLO band-structure with (b) the native minimalistic TB-model for the Ru-system and with (c) the Rh TB-model with Ru-like 4d-onsite energies.

For the Ru-system Figure S9 shows the comparison between the actual FPLO calculation, the native minimalistic TB-model derived from the Ru calculation and the Rh TB-model with altered Ru-like 4d-onsite energies. The three band-structures all show the important feature that the cone-forming p_z bands are separated by a gap from the higher lying bands. It shall be noted

here that the p_z bands near the Fermi energy contain $p_{x,y}$ and $4d$-character as well (hidden under the p_z weight). These bands are not pure Bi p_z-bands. In fact the development is such that the p_z-character decreases when going from Ru to Pd and Os to Pt. This can be understood from a simple model as discussed below.

An unfortunate fact is that the Bi $p_{x,y}$-orbitals heavily mix into the other bands, which makes it impossible to ignore them. A pure Bi-p_z, M-d model is very appealing in its simplicity but does not reproduce the qualitative features and trends of the series.

Owed to the complexity of the compounds the parameter space of even a minimalistic model is rather large. There are many possible ways of switching on interactions, some of which obfuscate the formation of the cones at the K-point. We chose to cluster interaction parameters into groups and to scale/shift the corresponding interactions simultaneously (e.g. we scale all interactions between Bi $p_{x,y}$ orbitals and all other orbitals by a single factor.)

Figure S10 shows some stages of the development of the band-structure with switching on of certain interactions. Figure S10 a) shows the starting point with Ru-like $4d$-onsite energies and full d-d and p_z-p_z couplings. All other couplings are zero. The p_z-framework bands (in red) discussed above are clearly visible. In b) we switched on all couplings except for $d-p_{xy}$ and $d-p_z$. As can be seen the p_{xy} bands do not form a nice gap, which maybe is not so surprising since graphene without a $2s$ orbital would have $2p_{x,y}$ bands crossing the Fermi energy. It is the sp^2 hybrid formation, which creates the huge gap between the bonding and anti-bonding $2p_{x,y}$ bands in graphene. In Bi the $6s$ bands are fully filled and hence cannot form sp^2 hybrids as is possible for carbon. However, the BL-anti-bonding triangle MO-bands at $+1$eV are clearly visible.

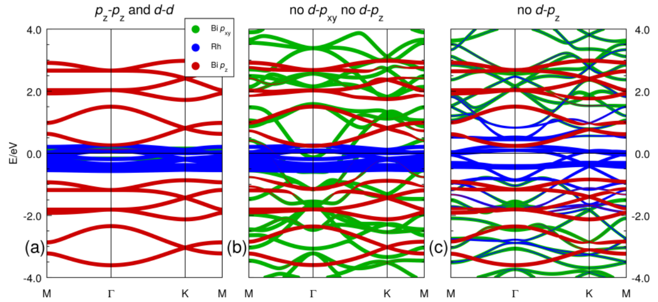

Figure S10. Model with Ru d-onsite energies for various couplings. Explanation in text.

When switching on the M d-$p_{x,y}$ interactions (Figure S10 c) the $p_{x,y}$ bands around the Fermi level become hybrids with the d-orbitals, which pushes the $p_{x,y}$ spectral weight away from the Fermi level. The MO-p_z bands are still discernible although the cone at the K-point got hybridised away. Instead we get two tiny cones of mixed character (Figure S11(a)).

At last, we slowly increase the coupling between the d-orbitals and the p_z-orbitals in a few steps. As shown in Figure S11, the $4d$-p_z coupling starts to separate the BL-AB MO bands. The lower one moves down and forms a new cone at the K-point with the help of $p_{x,y}$and $4d$ hybridisation, while all the time retaining the p_z character. In contrast the BL-bonding MO bands at -3eV stay rather untouched (not shown). This is certainly partially due to the energy separation between the $4d$-bands (band centre at around 0eV for Ru) and the bonding MO bands (at -3eV).

However, an insight can be gained from a simple 3-level system, which mimics the situation of the combined Bi-p_z M-d system. Let there be two levels at 0 with a BL interaction of $B = 2$ eV (Bi p_z-system) and one level at some energy Δ with a coupling to the two levels of $t = 0.4$eV (M-d-Bi-p_z coupling). The Hamiltonian is

$$H = \begin{pmatrix} 0 & B & t \\ B & 0 & t \\ t & t & \Delta \end{pmatrix}$$

and has the solutions shown in Figure S12(a). One peculiarity is that the bonding part of the 2-level (Bi) sub-system does not interact with the third level (M), which could be an additional reason for the absence of strong interaction between the d-system and the bonding MO in the TB-model discussed above. The Ru system corresponds to $\Delta = 0$ in this simple scheme, while Rh corresponds to $\Delta = -1.3$ and Pd to $\Delta = -2.5$. It is clear that the amount of Bi weight in the lower of the two hybridizing levels (blue) decreases when decreasing Δ (going from Ru to Pd), which is one reason why the Bi-p_z weight in the cone at the K-point decreases when increasing the nuclear charge and hence lowering the d-band centre (see Figure S13f).

In Figure S13 the M d-orbital onsite energies are shifted relative to the p_z band centre from $\Delta = 0.0$ (Ru) over $\Delta = -0.8$ (Ir) $\Delta = -1.3$ (Rh) to -2.3 (Pd,Pt). (These Δ-element correspondences are only approximately related to the parameters in Table S2, and chosen such that the band-structure around the cone in the resulting model most resembles the actual FPLO calculations.) Clearly the Dirac cone at the K-point stays in the gap but it's upper band reconnects with the conduction bands

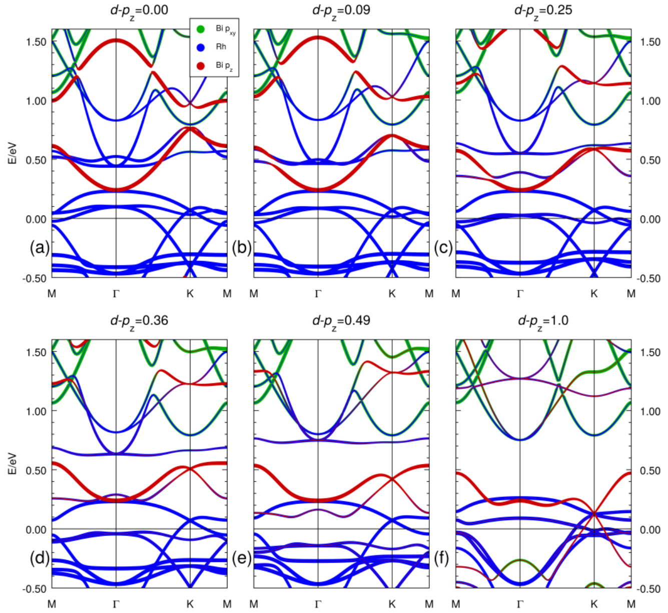

Figure S11. Model with Ru d-onsite energies switching on the final d-p_z coupling.

at the Γ-point. Interestingly the touching happens to occur for the Rh system, such that another "cone" with p_z character appears at the Γ-point. For $\Delta = -2.3$ (Pd) the p_z spectral weight has risen even further than the conduction band bottom, while the cone at the K-point starts immersing into the valence band continuum. In the process of tuning from "Ru" to "Pd" the BL-anti-bonding triangular MO bands around $+1$eV are restored again, while the bonding counterparts at -3eV are weakened since they fall into the M d-band centre. After all the 3-level model is rather simplistic. It however predicts that the energy position of the BL-bonding and anti-bonding MO bands stays rather constant in the parameter region applicable for our systems ($[-2.5\ldots0.0]$). This is illustrated in another series of pictures with the same parameter variation as in Figure S13 but this time the triangular MO spectral weight is shown. This also clearly shows that the cone at the K-point is not merely a shifted version of the MO-bands of Figure S8 but rather created through hybridisation with the other orbitals. Especially the $p_{x,y}$ system cannot be ignored. For instance for Ru parameters without the $p_{x,y}$ bands there would be no gap. A mechanism of how the bands forming the cone at the Fermi level come about is sketched in Figure S12(b). If the BL levels have dispersion the third level (d-orbital) will lead to (modified) replicas of the BL bands at energies corresponding to the 3-level spectrum.

The evolution of the system discussed above shows how the graphene like part of the simple Bi p_z framework band-structure turns into the cones observed in the scalar-relativistic band-structures of our series of systems. The cone is a consequence of several hybridisations but essentially contains spectral weight of the MO, which clusters 6 Bi p_z orbitals together. Because of this one can speculate that the similarities with graphene also transfer to the topological properties of the system.

Finally, as an illustration we show the real space picture of a band with BL-triangular MO weight at the Γ-point in Figure 4d.

The topology

Based on the analysis of the band-structure we come to the conclusion that the Dirac-cone occurring at the K-point in a calculation without spin orbit coupling is related to an effective molecular orbital which combines 6 Bi $6p_z$ orbitals into a pseudo-carbon p_z orbital . Because of the known topological properties of the graphene lattice, we speculate that the topological nature of the Bi-M-I compounds is similarly related to the lattice arrangement of the pseudo p_z MO. This would indicate that the Dirac-cones must evolve into a gapped band-structure under inclusion of spin-orbit coupling. Indeed we observe that the cones follow this scheme although in a more complicated matter. One complication arises from the hybridisation with other bands which leads to the fact that the topological active bands need not be the bands, which form the cone. Topological active we call bands, for which formally the Z_2 invariant switches from trivial to non-trivial or back provided it was the last band below a gap. Of course this is not always given. One can formally calculate the invariants using the Fu&Kane formula for inversion symmetric systems. If the last band for which we multiply the parities is separated by a gap from higher bands, the phase continuity condition for all bands below can be fulfilled and the thus calculated invariant is correct. If the last included band is not separated by a gap from bands above, we still calculated the parity changes but the phase continuity condition can be fulfilled after a few bands above the last included if a gap opens above those bands. In such a case the topology effectively

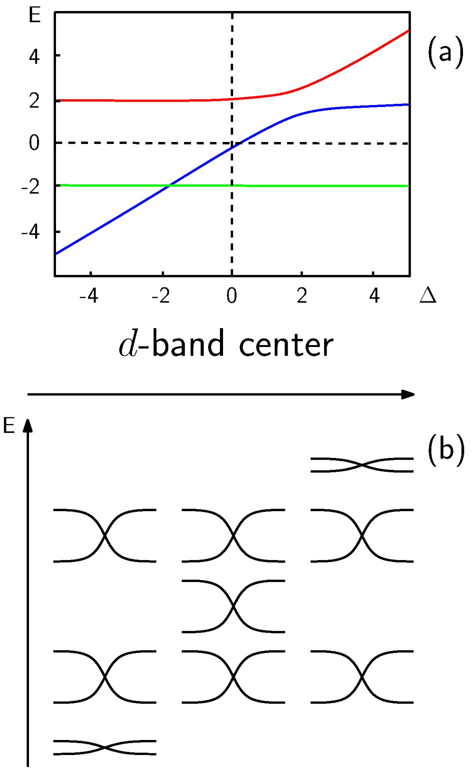

Figure S12. (a): the spectrum of the 3-level system d-system onsite energy Δ. (b): schematics of a three level system with additional dispersion.

changes at the lower gap boundary although the band inversions/parity changes happened several bands below the gap. With this definition of topological active bands we can at least mark those bands above which the next gap will have changed topology. It turns out that in the Bi-M-I-systems many topologically active bands have Bi p_z character. Of course in such a multi bands system there is no reason why other bands could not be topologically active.

The series of compounds can now be understood by observing the bands with Bi p_z spectral weight around the Fermi level. By tuning the spin-orbit coupling to a small value one can compare the full relativistic bands with the nearly scalar-relativistic bands. Figure 3 is a sketch of the most distinguished cases. For the Os and Ru compounds the two bands forming the cone at the K-point both are topologically active. Above the higher of the two bands there is a gap. With increasing SO coupling the cone develops into a gap and two non-crossing topologically active bands develop. The first switches to non-trivial and the second back to trivial. Since there is a gap above the second band the topologically non-trivial energy region is quite narrow. In fact the two bands are so close that no real gap opens. Instead we just have non-crossing bands forming a gap “warped in k-space”. The next compound in the order of bonding features (explained in Sec. *The tight-binding description*) would be Ir. In this case the cone forming bands cover a larger energy region. Yet there still persists an energy gap above the cone forming bands. Increasing SO coupling opens the cone and two topologically active bands form. Owed to the larger energy spread of the original cone bands a real topologically non-trivial gap appears between the two bands.

This brings us to the remaining three compounds Rh, Pt and Pd. They are essentially different in that the cone forming bands spread so widely that a second band crossing (cone-like) happens, this time at the Γ-point. This second cone allows for a second band inversion to take place at the Γ-point cone, which now means that the lower band forming the cone at K is active but the upper one is not. Instead, the bands forming the upper part of the Γ-point cone are topologically active. Now, SO coupling will gap both cones without changing the “activeness”, which results in a parasitic topologically in-active band in a large gap between active bands stemming from the lower part of the K-cone and the upper part of the Γ-cone. This is the origin of the large non-trivial gap for the Rh, Pt and Pd compounds. In fact it seems that the Rh system is just at the verge of this scenario for Rh (Sec. *The tight-binding description*). The p_z spectral weight is moving to higher and higher energy in the conduction bands when going from Rh to Pt and Pd. At the same time the same weight in the occupied sector moves lower, which results in more and more bands being parasitic bands within a large non-trivial gap along this series.

To conclude, for a large topological gap with possibly several trivial (parasitic) bands within the gap a double cone structure at the K and Γ-points is needed, which in turn depends on the position of the Bi p_z anti-bonding spectral weight, which evolves

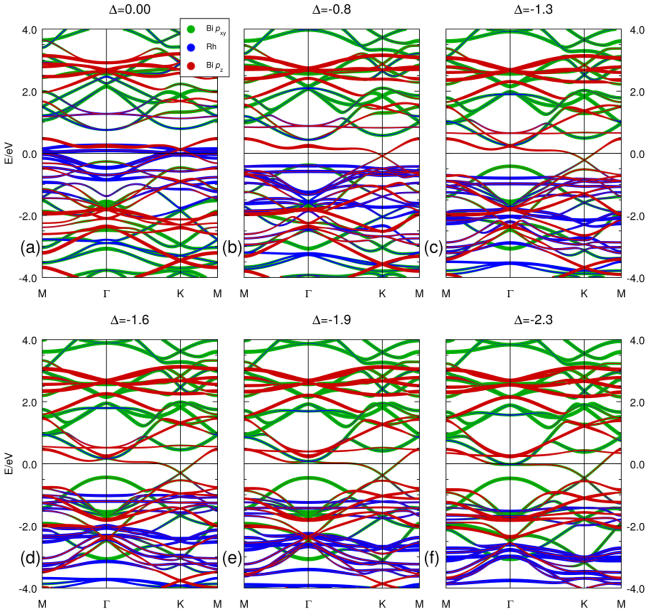

Figure S13. Model with varying onsite energies. $\Delta = 0.0$ corresponds to Ru, $\Delta = -0.8$ to Ir, $\Delta = -1.3$ to Rh and $\Delta = -2.3$ to Pd.

favourably with a downward shift of the M d-band centre.

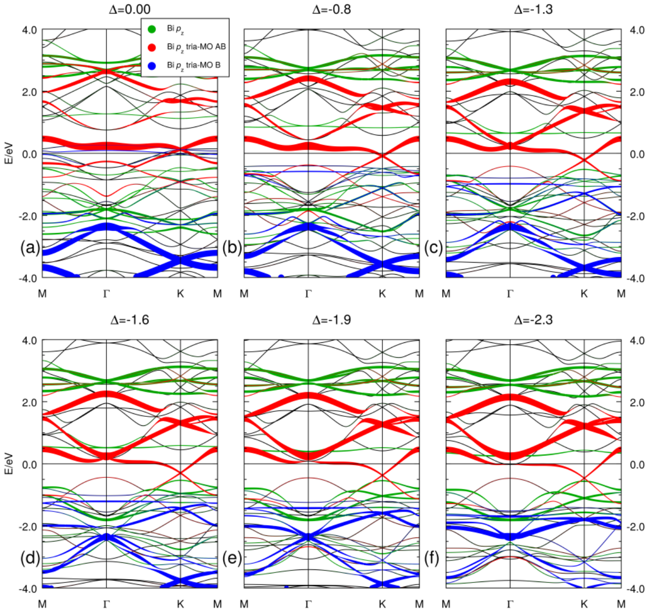

Figure S14. Model with varying onsite energies. The spectral weight of the BL-bonding and anti-bonding triangular MO is shown.
